# Understanding Post-Adoption Behavioral Intentions of Mobile Health Service Users: An Empirical Study during COVID-19

**DOI:** 10.3390/ijerph20053907

**Published:** 2023-02-22

**Authors:** Yanmei Jiang, Antonio K. W. Lau

**Affiliations:** 1 The School of Business, Anhui University of Technology, Ma’anshan 243032, China; 2 Key Laboratory of Multidisciplinary Management and Control of Complex Systems of Anhui Higher Education Institutes, Anhui University of Technology, Ma’anshan 243032, China; 3 The School of Management, Kyung Hee University, Seoul 02447, Republic of Korea

**Keywords:** mobile health, cognitive trust, emotional trust, continuance intention, positive WOM, COVID-19

## Abstract

This study aims to understand the post-adoption behaviors of mobile health (m-Health) service users during the COVID-19 pandemic. Drawing on the stimulus-organism-response framework, we examined the effects of user personality traits, doctor characteristics, and perceived risks on user continuance intentions and positive word of mouth (WOM) when using m-Health, as mediated by cognitive and emotional trust. The empirical data were collected via an online survey questionnaire from 621 m-Health service users in China and were verified with partial least squares structural equation modeling. The results showed that personal traits and doctor characteristics were positively associated and the perceived risks were negatively associated with both cognitive and emotional trust. Both cognitive and emotional trust significantly influenced users’ post-adoption behavioral intentions in terms of continuance intentions and positive WOM, with different magnitudes. This study provides new insights for the promotion of the sustainable development of m-Health businesses after or during the pandemic.

## 1. Introduction

The outbreak of COVID-19 has provided unprecedented impetus to the development of the mobile health (m-Health) services that have been widely adopted to mitigate the pandemic problems with real-time information dissemination, remote medical consultation, reduced medical costs, and minimized exposure and interpersonal cross-infection, as well to improve user health management, such as with self-assessment, telemedicine-based consultation, and contact tracing [1,2,3]. m-Health services could enhance user medication engagement and the self-management of healthcare knowledge from the perspectives of patients and healthcare professionals [4]. m-Health services can be defined as “*healthcare to anyone, anytime, and anywhere by removing locational, time, and other restraints while increasing both the coverage and the quality of healthcare*” ([5], p. 50). For example, it uses mobile computing technologies such as smartphones for healthcare use [1]. As relatively new technologies, m-Health services are mainly examined in the recent literature from the perspectives of usability assessment, adoption of the new technology, and healthcare policy support, but a few studies provide empirical support for the sustainable development of m-Health services from the perspective of individual users during the COVID-19 context [3,6,7].

While the users’ adoption intentions and their influencing factors are the focus of the existing literature [8,9,10,11,12,13], a few scholars examine the users’ post-adoption behavioral intention for m-Health services [14,15,16], especially with regard to the continuance intention and the positive word of mouth (WOM) after use. The individual’s continuance intention is closely associated with the repeated use of m-Health, while the users’ positive WOM not only reflects their own attitudes and behavioral intentions towards a certain m-Health platform, it also plays a non-negligible role in influencing the potential users of m-Health services. Although both are critical for the sustainable development of m-Health services, there is very little research on continuance intention and positive WOM based on the users’ experiences during the pandemic context [17,18].

The development of m-Health services involves three main actors: the individual users, the doctors that provide online consultation services to the users, and the m-Health platforms. For the users, their personality traits, such as whether they have a propensity to trust the use of new technologies, have a crucial effect on their behaviors as [19,20]. The ability and the benevolence of the doctors are relevant to the doctor–patient relationships that affect reuse intention [10]. The management of the m-Health platforms plays a significant role in the effective operations of m-Health services [17] and in developing consumer trust [21]. In fact, the recent studies show that the use of m-Health applications could improve patient satisfaction in terms of convenience, health information sharing, and the transparency of medical service fees [22]. Individual users rely on cognitive and emotional trust when making decisions [10]. However, their distinctive impact on the m-Health service users’ post-adoption behavioral intentions during the pandemic context is rarely reported [10,15,21]. The effects of the relationships between the users’ personality traits, the doctors’ abilities and benevolence, and the emotional and cognitive trust in the m-Health platforms on the users’ behavioral intentions have not yet been explored in the literature.

Furthermore, with the rapid expansion of mobile medical application/platform markets, the risks of using m-Health services cannot be ignored, especially the privacy and physical risks [21,23]. When users attempt to access healthcare resources via an m-Health application, they must provide private and sensitive information about themselves and their immediate families. Privacy leakage is undoubtedly one of the most prominent risks of m-Health operations [21,24]. Moreover, when doctors and patients exchange information mainly through text messages, voice messages, or videos rather than face-to-face communication, this exchange process is more likely to result in misdiagnosis, causing physical risks to the users [25]. However, few scholars have examined their separate roles in m-Health services. Based on the literature above, this study proposes the following research question:

***What effects do user personality traits, doctor characteristics, and perceived risks have on user post-adoption behavioral intentions via cognitive trust and emotional trust in the m-Health service context?***

To answer the research question, we adopt the stimulus-organism-response (S-O-R) framework to study the effects of user personality traits, doctor characteristics, perceived risks, and user trust in the m-Health platform on continuance intention and positive WOM. User trust includes cognitive trust and emotional trust, which will be distinctively examined in the m-Health platform with regard to the users’ continuance intentions and to positive WOM in the pandemic situation.

This research has three contributions. First, this study enriches the existing literature on m-Health services by comprehensively considering the three major actors of m-Health services [20]: the individual users, the doctors, and the platforms. Our research also advances the existing literature on online trust by investigating the distinct effects of cognitive and emotional trust in the m-Health service context, which are less explored in the literature [10,15]. Second, this study examines the antecedents of continuance intention and positive WOM and their interrelationships in m-Health situations by studying them simultaneously in a single model. This may help m-Health companies effectively employ different business strategies to promote the use of m-Health services [26,27,28]. Finally, this study provides new evidence for the m-Health literature by augmenting the internal linkage mechanisms of stimulus (i.e., user personal traits, doctor characteristics, and perceived risks), organism (cognitive and emotional trust in the m-Health platform), and responses (i.e., user behavioral intentions) in the S-O-R framework. The results can enrich our knowledge of how the different actors play their roles in affecting users in their continued (re-)use of online health services, which, if properly promoted, may effectively reduce medical costs for the post-COVID-19 period [2,3].

## 2. Conceptual Framework

### 2.1. S-O-R Framework

**Figure 1:**
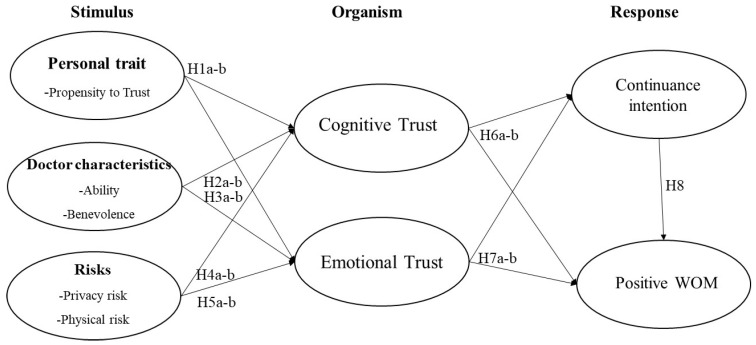
Research model.

The S-O-R framework was proposed by [29,30]. This framework suggests that the external environment and personal traits can act as external and internal stimuli that trigger an individual’s cognitive process and emotional state. Such an organismic experience then results in a series of behavioral responses. The S-O-R framework has been widely applied in academic fields, such as retailing, hospitality, online shopping, and social commerce [31,32,33,34]. For instance, following the S-O-R framework, Cho et al. [35] suggested that autonomy interactivity, self-expression, and visual aesthetics as stimuli can affect the technological and aesthetic aspects of user satisfaction and pleasure (O), which subsequently impact product attachment (R) in e-commerce. Brewer and Sebby [36] investigated the effects of menu appeal, menu informativeness, and perception of COVID-19 (S) on consumer purchase intention (R) through the desire for food and the perceived convenience of online food ordering (O). In the m-Health context, continuance intention (R) can be affected by health empowerment (O) and gratification (O) with perceived affordances (S) [37]. Information, the system, and the service quality (S) affect user engagement, satisfaction, and the love of the app (O), leading to continuance intention, WOM, and stickiness intention (R) with regard to a mobile fitness application [38]. Chudhery et al. [39] explores the ways in which the technology-based m-Health characteristics (S) affect initial trust and satisfaction (O), resulting in behavioral intention to use the m-Health services (R). Unlike those studies, this study adopts the S-O-R framework to first examine how the trust from patients and doctors (S) affects two different types of trust in m-Health apps/platforms (O) with regard to continuance intention and positive WOM (R) in online medical consultations. Accordingly, this study applies the S-O-R model to understand m-Health service users’ post-adoption behavioral intentions by simultaneously considering the internal and external stimuli. More specifically, we regard personal traits as the internal stimuli and doctor characteristics and the physical and psychological risks as the external stimuli. The users’ cognitive trust and emotional trust are regarded as the organism. Finally, the users’ post-adoption behavioral intentions (i.e., continuance intention and positive WOM) are the responses in the model, as shown in Figure 1.

In addition, the recent literature also examines the continuance intention in m-Health through different frameworks and models [40]. For example, adapting information systems continuance and success models, Song et al. [41] explores the roles of perceived health status, usefulness, and user satisfaction in continuous use intention. Kaium et al. [42] explores m-Health with a unified theory of acceptance and the use of technology models. Using the elaboration likelihood model, Hsiao and Chen [43] shows that the social media influence affects a patient’s attitude toward m-Health services. The expectation–confirmation model was adopted to examine how m-Health continuance intention is affected by perceived usefulness, technology maturity, individual habits, task mobility, and use satisfaction [44] or by perceived risk, perceived interactivity, and facilitating conditions [27]. Using service quality frameworks, Kim et al. [45] shows that engagement and satisfaction are critical for continuance intention. Kim and Han [46] applied social cognitive models to examine the roles of regularity behavior, outcome expectation, safety efficacy, and privacy risk on continuance intention. Other recent studies include [14,15,47,48].

### 2.2. Cognitive Trust and Emotional Trust

Trust is important to any personal or organizational relationship [10,49,50] as it helps individuals to overcome uncertainties and risks [20,51]. Trust decisions involve both reasoning and feeling aspects, which can be understood as cognitive trust and emotional trust, respectively [49]. In consumer service relationships [52], cognitive trust refers to the confidence and willingness to rely on a service provider’s ability and reliability, whereas emotional trust is the willingness and confidence one places in a service provider based on the emotional bonds between them. Cognitive and emotional trust are highly correlated but have distinct functions in the individual decision-making process [10]. For example, drawing on the social exchange and information processing theory, Lu et al. [53] showed the positive relationships between internet health information quality, the source of internet health information, and patient compliance via cognitive and affective trust. Based on the online trust framework, cognitive and emotional trust are found to separately mediate the relationship between the physicians’ attributes and the patients’ willingness to choose [10]. Following the existing literature, this study aims to investigate the roles of cognitive and emotional trust in the m-Health service context and to explore their distinct effects on the users’ post-adoption behavioral intentions in the pandemic context.

## 3. Research Model and Hypotheses

Based on the S-O-R framework, we propose a research model, as shown in Figure 1, to demonstrate that an individual user’s continuance intention and positive WOM (responses) can be influenced by an individual’s personality traits, the doctor’s characteristics, and the risks (stimuli) through cognitive trust and emotional trust (organism) in the context of the m-Health service. The following hypotheses are developed accordingly.

### 3.1. Antecedents of Cognitive and Emotional Trust in the m-Health Platform

A disposition to trust refers to an individual’s general willingness to trust or depend on others [51]. It is the result of ongoing lifelong experiences and socialization [51,54] and is stable over time. It is the general belief that other people are usually well-meaning and reliable [55]. A person with a higher propensity to trust is more likely to trust others [56], especially in an unfamiliar situation. In other words, when people need to assess or make decisions during the initial phases of a relationship, those with a greater propensity to trust are more likely to trust others [57,58]. This personal trait can color an individual’s interpretations of events and behaviors in a relationship, influencing the development of user trust [51,59]. For instance, citizens with a greater propensity to trust may prefer to trust others in the context of e-government [60]. Park and Tussyadiah [61] estimated the effects of a disposition to trust on trusting beliefs consisting of cognitive and emotional components. In the same vein, we believe that individual users with a higher propensity to trust would be more likely to trust the m-Health platform cognitively and emotionally. Thus, the following hypotheses are proposed:

**H1a.**  *The propensity to trust is positively associated with the cognitive trust in m-Health platforms*.

**H1b.**  *The propensity to trust is positively associated with emotional trust in m-Health platforms*.

In the m-Health services, the doctors’ abilities and benevolence in treating their patients are important factors for the users when seeking consultation via an online platform [62,63]. An ability is one’s skill and competence in a specific domain [64]; so, the doctors’ abilities refer to their titles, qualifications, and practical competence in the relevant field. In the m-Health service context, the doctors’ genders, affiliations, titles, and pictures can be found on the mobile platform, which provides basic information and knowledge about doctors and the m-Health platforms for the users. The information improves the users’ understanding of the doctors, which can directly enhance the users’ cognitive trust in the m-Health platform [62]. A doctor’s practical competence can be judged through the doctor’s diagnoses and treatment records, as well as the frequent interactions between the physician and the patients, whose activities should improve emotional trust [65]. For instance, in the online health consultation context, patients’ online trust can be affected by the physicians’ abilities, which consist of professional knowledge, physician rank, treatment effect, and physician image [10].

Benevolence refers to a sincere concern for the interests of others and the motivation to do something good for others [51,62]. In the current research context, the benevolence of doctors means that the doctors care about their patients and are motivated to act in the patients’ interests [10,62]. A doctor’s benevolence towards patients can be manifested in the doctor’s good service attitude and communication skills [10,65], such as listening to patients’ feelings and showing genuine concern. Because these activities convey concern, warmth, and friendliness to patients, they strengthen the trust relationships between users, doctors, and m-Health platforms. Good communication between doctors and patients affects doctor–patient trust by helping both sides to build good impressions and to eliminate the psychological barriers between them [66]. Thus, the following hypotheses are proposed:

**H2a.**  *A doctor’s ability is positively associated with cognitive trust in m-Health platforms*.

**H2b.**  *A doctor’s ability is positively associated with emotional trust in m-Health platforms*.

**H3a.**  *A doctor’s benevolence is positively associated with cognitive trust in m-Health platforms*.

**H3b.**  *A doctor’s benevolence is positively associated with emotional trust in m-Health platforms*.

Perceived risk is considered a multifaceted concept in e-commerce and online platform literature [20,67,68,69], and we suggest that it is crucial to examine the privacy risk and the physical risk in the m-Health platforms. Privacy risk refers to the probability of having personal information disclosed [70] as the result of using an m-Health service, whereas physical risk refers to the potential risk of physical injury when using it [71]. To use m-Health services, users need to provide very sensitive personal health-related information in addition to the general personal data required for registration, creating a high risk of losing their private information to outsiders [21]. In addition, since online consultation is not conducted face-to-face but through text and voice, there may be more miscommunication between doctors and patients, resulting in misdiagnosis and the delaying of treatment, posing a physical risk to patients [67].

While information technology provides users with personalized services, it increases the risk of personal information leakage [21], which makes potential users hesitate to participate in m-Health services. The previous research shows that the users who are concerned about privacy are less likely to trust m-Health service providers, which can reduce their intention to use the service [21,72]. Conversely, the users’ trust will increase if they perceive a low risk in using an online service or product [16]. Tang et al. [73] found that the users’ trust in online medical websites decreased if they perceived a high level of physical or privacy risk. Therefore, the following hypotheses are proposed:

**H4a.**  *Privacy risk is negatively associated with cognitive trust in m-Health platforms*.

**H4b.**  *Privacy risk is negatively associated with emotional trust in m-Health platforms*.

**H5a.**  *Physical risk is negatively associated with cognitive trust in m-Health platforms*.

**H5b.**  *Physical risk is negatively associated with emotional trust in m-Health platforms*.

### 3.2. Roles of Cognitive and Emotional Trust in m-Health Platforms

Trust plays an important role in user behaviors [16,20,52,74,75] and consists of cognitive and emotional dimensions [49]. Cognitive trust and emotional trust are two essential components of consumer decision making [53], which relate to each other with distinctive functions [49]. In this study, cognitive trust can be understood as the users’ confidence in relying on an m-Health platform, based on its ability and reliability, while emotional trust refers to the users’ willingness to use the platform based on their emotional connections [15,52].

The previous studies show that user trust positively influences behavioral intentions, such as continuance intention and positive WOM [20,76,77,78]. In the m-Health service context, [15] showed that both emotional and cognitive trust can positively affect the users’ continuance intentions when using m-Health services (see also [53]). Patients’ cognitive and emotional trust improved their behavioral intentions in the online health consultation context [10]. In addition, the previous studies identified trust as an important antecedent of WOM [79]. The higher the level of consumer trust, the higher the level of positive WOM [61]. Thus, the following hypotheses are proposed:

**H6a.**  *Cognitive trust in m-Health platforms is positively associated with continuance intention*.

**H6b.**  *Cognitive trust in m-Health platforms is positively associated with positive WOM*.

**H7a.**  *Emotional trust in m-Health platforms is positively associated with continuance intention*.

**H7b.**  *Emotional trust in m-Health platforms is positively associated with positive WOM*.

### 3.3. Relationship between Continuance Intention and Positive WOM

The previous literature shows that continuance intention can generate positive WOM behaviors [26,27]. When consumers have continuance intentions for a given m-Health platform, they will be more likely to recommend this platform to their friends and relatives; to be more engaged in processing new information about the platform; and to be greatly resistant to the persuasion of contrary information [79]. The relationship between continuance intention and positive WOM has been empirically verified in different research contexts, such as mobile internet-based health services [27], online travel services [28], and e-banking operations [26], but not in the m-Health context. Following the existing literature, we suggest that the users’ continuance intentions are related to positive WOM in the m-Health services context. Thus, the following hypothesis is proposed:

**H8.**  *Continuance intention is positively associated with positive WOM*.

## 4. Methodology

### 4.1. Research Approach

In this study, an online survey research approach was adopted for two reasons. First, research on the impact of personal trust and doctor attributes on health mobile platforms and the role of consumer trust in continuance intention and WOM requires conducting a self-reported survey to measure the consumers’ feelings and experiences. Second, an online survey is suitable for the study of online platforms, and it is especially suitable for our study during the COVID-19 period to avoid face-to-face discussions and possible COVID-19 infections. To analyze the collected data, partial least square structural equation modeling (PLS-SEM) was used due to the complexity of the research model [80,81].

### 4.2. Research Context

In China, m-Health services were mainly conducted between patients and healthcare service providers through m-Health platforms/applications. The platforms functioned as intermediators to coordinate patients with health service providers such as hospitals and clinics and to process transactions. The platforms promoted, sold, facilitated, and delivered healthcare services, including online medical consultations, the scheduling of hospital visiting or examination activities, the delivery of medicines, the selling and claiming of insurance, and health information management. Healthcare service providers such as hospital doctors provided online consultation services to the m-Health patients as independent contractors, while working full time in the public hospitals at the same time. A few m-Health platforms might set up internet-based hospitals for online consultations. In China, there were several major m-Health platforms that provided online consultations, i.e., Haodf.com, WeDoctor, Chunyu Doctor, Ping An Good Doctor, AliHealth, and JD Health [82,83], which were included in our study. Table 1 shows their basic information and functionalities.

### 4.3. Measurements

We adapted the measurement scales from the existing literature for the current context. The questionnaire items used a seven-point Likert scale ranging from strongly disagree (1) to strongly agree (7). The scales of propensity to trust were adapted from [61]. The measurements of benevolence and ability were borrowed from [61,84]. The measurements of privacy risk and physical risk were adapted from [73,85]. The scales of cognitive trust and emotional trust were adapted from [49,53]. The measurements of continuance intention were adapted from [86,87]. The positive WOM measurements were adapted from [88,89,90]. To measure the continuance intention and WOM, we referred our questions to the m-Health application platform. For example, we stated “*I intend to continue using this app in the future*” (i.e., ContinuanceIntention1). As discussed above, the m-Health app in the studied context functioned as a platform with which the consumers consulted with the doctors via the app but not directly through the hospitals or clinics. Thus, focusing on our study objective, we intended to understand how the patients would trust the app to and continue to use its services.

### 4.4. Survey Design and Data Collection

The survey questionnaire consisted of four parts. In the first part, we described the purpose of the study and declared that the data collection was anonymous and used only for academic research. To ensure the appropriateness of the respondents, screening questions were placed in the second part, including those on past experience with paying for the online consultation services of m-Health applications, the name of the most frequently used platform, and the frequency of use. Thus, our study is different from the other existing research that focused on the early adoption of the m-Health service (e.g., [12]). As discussed above, m-Health application could provide several healthcare services for the customers. When our model involved doctor characteristics and physical risks as key factors impacting upon the trust and continuance intentions regarding the m-Health app, the online consultation service was selected. This was because we believed that during the online consultation patients had to interact with the designated doctor and would likely be exposed to higher immediate risks following the doctor’s advice on medical treatments than when dealing with health management and education, reading information about hospitals and doctors, or completing patient satisfaction surveys. The research constructs were contained in the third part. The final section included the demographic information of the respondents. We drafted the initial questionnaire in English and then translated it into Chinese. A back-translation method, a set of pilot tests that interviewed seven Chinese graduate students, and a pretest of 67 Chinese consumers who had experienced online consultation using m-Health applications were conducted to verify the questionnaire’s content.

A research company called Wenjuanxing, which had over 6.2 million registered members in China [91], was employed to help in collecting the data. The current research study has recruited this company for consumer trust studies (e.g., [20]). The online survey questionnaire was randomly distributed to 700 members by the survey company from 31 January 2021 to 8 February 2021, during the COVID-19 pandemic period. We selected the respondents who had paid for the online healthcare consultation services of m-Health applications in the previous 12 months. After removing the invalid responses (e.g., not meeting the screening criteria or uncompleted responses), a total of 679 valid data items were used to conduct the data analysis. To ensure that there was sufficient power to analyze the data, a post hoc statistical analysis was conducted [92] using [93] a post hoc statistical power calculator. The power analysis considered 1–β as a function of significant level α, sample size, and observed R^2^. The results showed that the observed statistical power of positive WOM, continuance intention, cognitive trust, and emotional trust was equal to one, indicating that the study had adequate power (>0.85) [94].

### 4.5. Sample Profiles

The sample profiles are reported in Table 2. In the sample, 379 (55.8%) were females and 300 (44.2%) were males. Most of the participants were between 31 and 40 years old. The majority of the respondents were married (76.1%). Regarding income level, 85.8% reported their monthly salary as more than RMB 5000. Regarding education level, most of the respondents had an undergraduate degree. In the sample, Ping An Good Doctor was the most used m-Health application in the previous 12 months (45.4%), followed by Good Doctor (21.1%), and Ding Xiang Doctor (20.2%). This result was consistent with the existing research [82], which states that most of the users of m-Health services in China are between the ages of 26 and 35, are married, and have a higher education degree and a stable income. These users were more willing to adopt new technologies to improve their lifestyles, to reduce the risks of possible diseases, and to keep abreast of their own or their families’ health status. Tian and Wu [95] also noted that m-Health in China was not popular for elderly patients. Finally, regarding use frequency, most participants (85.7%) reported using m-Health applications more than twice last year, with the larger group (57.7%) using m-Health apps two or three times.

## 5. Results

### 5.1. Measurement Model

A two-stage analysis approach was adopted to analyze the data [81]. First, we assessed the reflective measurement model, which involved the evaluation of the reliability, the convergent validity, and the discriminant validity of the research constructs. For internal consistency reliability, as shown in Table 3, all of the Cronbach’s α values of the constructs were higher than the acceptable threshold value of 0.6 [96,97], with most of them above the satisfactory threshold value of 0.7. All of the composite reliability (CR) values of the constructs were greater than the suggested value of 0.7. These results implied a good internal consistency reliability in the measurement model [81]. Regarding the convergent validity, all of the average variance extracted (AVE) values of the constructs were between 0.519 and 0.856 and were greater than the threshold value of 0.5, indicating a satisfactory convergent validity of the model.

Regarding discriminant validity, the cross-loading criterion and the Fornell–Larcker criterion were adopted. The former dealt with the indicator level, and the latter dealt with the construct level [66]. Table 4 shows that all the loadings of the indicators in each construct were greater than the cross-loading of the indicators in the other constructs. Table 5 shows that all the square roots of each construct’s AVE were greater than the correlations with the other constructs. These results suggested that the discriminant validity of the measurement model was established.

We also conducted the heterotrait–monotrait (HTMT) ratio analysis to further assess the discriminant validity [98]. The HTMT is the ratio of the between-trait correlations to the within-trait correlations [66]. Table 6 shows that all the HTMT ratios were lower than the threshold value of 0.9 [98], indicating a satisfactory discriminant validity in this study.

### 5.2. Common Method Bias Assessment

The common method bias was initially assessed using Harman’s one-factor test. The results showed that the single factor accounted for 26.997% of the variance, suggesting that the bias in this study was not very serious. Consistent with [80,99], a full collinearity test was then adopted to evaluate the bias. The results indicated that the inner variance inflation factor (VIF) values were between 1.062 and 1.994 and were lower than the suggested value of 3.3 [100]. These results also suggested that bias was not a major concern in this study.

### 5.3. Structural Model

After the measurement model was confirmed, we evaluated the structural equation model, as recommended by the PLS-SEM studies [66,101], which included tests of the goodness of fit, the path coefficient, the effect size (F^2^), the coefficient of determination (R^2^), and Stone–Geisser’s prediction relevance (Q^2^).

#### 5.3.1. Goodness of Fit

The standardized root mean square residual (SRMR) was the root of the mean square discrepancy between the observed correlations and the model-implied correlations [66], and the SRMR value should be less than the threshold value of 0.08 for a good fit model. Our results showed that the SRMR value of the structural model was 0.061, far less than the suggested value. Moreover, the root mean square residual covariance (RMS_theta) was used as another way to measure model fitness. RMS_theta followed the same logic as the SRMR but depended on the covariances [66]. Our results showed that the RMS_theta value of the structural model was 0.114, which was less than the threshold value of 0.12 [102]. These results indicated that our structural model was a good fit.

#### 5.3.2. Path Coefficient

A bootstrapping procedure in SmartPLS with 5000 subsamples was adopted to examine the significance of the hypothesized relationships in this study. As shown in Table 7, the propensity to trust significantly affected the consumers’ cognitive and emotional trust in m-Health applications, supporting H1a and H1b. The doctor’s ability was positively associated with cognitive and emotional trust, supporting H2a and H2b. The doctors’ benevolence significantly influenced both cognitive and emotional trust, supporting H3a and H3b. A comparison of the coefficient values of these two doctor characteristics showed that the doctor’s benevolence could have a stronger effect on both emotional and cognitive trust than the doctor’s ability. The perceived privacy risk and physical risk negatively affected cognitive and emotional trust; so, H4a and H4b and H5a and H5b were accepted. Moreover, both cognitive and emotional trust significantly affected continuance intention and positive WOM, supporting H6a and H6b and H7a and H7b, respectively. Finally, continuance intention significantly affected positive WOM; so, H8 was accepted. It is noted that the frequency of use of the m-Health app might affect the trust measures as frequent users have greater trust in the application. Thus, we added the frequency of use of the m-Health application as a control variable and re-ran the whole statistical analysis. All the statistical results, including the path coefficients and model fits, did not show any significant changes, which further supported our findings.

#### 5.3.3. Coefficient of Determination (R^2^)

The coefficient of determination (R^2^) was commonly used to measure the model’s predictive power, with a higher level of R^2^ indicating a good predictive accuracy of the model [66]. As depicted in Table 8, R^2^ and the adjusted R^2^ ranged from 0.337 to 0.514, indicating that our model had good predictive power.

#### 5.3.4. Effect Size (f^2^)

The change in the R^2^ value when a specific exogenous construct was omitted from the original model could be used to evaluate whether the omitted construct had a substantive effect on the endogenous constructs [66]. As shown in Table 9, the f^2^ value ranged from 0.01 to 0.143, indicating that most of the exogenous constructs had small or medium effects on the endogenous constructs [103].

#### 5.3.5. Prediction Relevance (Q^2^)

Stone–Geisser’s Q^2^ value was used to measure the predictive relevance of the model. In the structure model, Q^2^ values higher than zero indicated that the path model had predictive relevance for a specific endogenous construct [66]. Table 10 shows that all the Q^2^ values were considerably larger than zero, providing clear support for our model’s predictive relevance.

## 6. Discussion

Using to the S-O-R framework, this study investigates the factors influencing two different post-adoption behavioral intentions of m-Health service users (e.g., continuance intention and positive WOM). Consistent with the existing literature [57,59,61], our results show that an individual’s propensity to trust is positively associated with both cognitive and emotional trust in the m-Health platform, indicating that individual users with a greater propensity to trust would be more likely to trust the m-Health platform cognitively and emotionally.

Consistent with our expectations, a doctor’s benevolence positively affects the users’ cognitive and emotional trust in the m-Health platform, meaning that a doctor’s good service attitude and communication skills can play a significant role in developing the users’ trust during the online consultation process [10,50]. The ability of a doctor can do the same. However, we further explore the fact that the doctor’s benevolence has a stronger effect than the doctor’s ability on the users’ cognitive and emotional trust. This implies that the users’ trust in the platform can be largely affected by the users’ emotional connection with the doctor via the doctor’s kindness, goodwill, and friendly attitudes during their interaction [15,51,52]. While the doctor’s ability to treat the users cannot be ignored, it seems to show us that the doctor’s helpful behaviors toward the users are more crucial to the trust of m-Health services than the doctor’s ability. It is the extra caring behavior that a doctor displays for the users to improve their comfort that allows the users to cognitively and emotionally trust the platform.

Consistent with our hypotheses, both privacy and physical risks negatively affect the users’ trust cognitively and emotionally, indicating that both play a negative role when developing the users’ trust in m-Health platforms [16,21,23]. The privacy risk was found to have a greater negative effect on emotional trust, while physical risk had a greater effect on cognitive trust. This may be understandable as physical risk can refer in this study to misdiagnosis or delay in treatment, which is more dependent on the competence and ability of the service providers [52], while the privacy risk can be less related to the ability of a given m-Health platform and more about emotional connection.

As expected, both cognitive trust and emotional trust are positively associated with user continuance intention and positive WOM. Cognitive trust has a stronger effect on continuance intention, while emotional trust plays a more effective role in increasing the users’ positive WOM [15,59,104]. This result can be interpreted by the fact that when users intend to continue to reuse their m-Health platforms, they may pay more attention to cognitive trust in the platforms, which emphasizes the functions and performance of the m-Health applications. When users have an emotional connection with the platform, possibly due to the kindness of the doctors on the platform, they may positively promote it. Therefore, to encourage users to reuse m-Health services, m-Health service operators might improve the functions of the m-Health applications. On the other hand, if m-Health practitioners want to promote positive WOM to potential users, they could focus more on developing emotional connections with the existing users.

Finally, we helped to clarify the relationship between continuance intention and positive WOM in the m-Health service context. The results show that continuance intention is positively related to positive WOM, suggesting that continuance intention is a crucial but separate element with a positive WOM. This result is in line with the existing literature [26,27] that continuance intention and positive WOM are different user post-adoption behavioral intentions, but they are significantly correlated with each other.

## 7. Conclusions

This study shows that there are many personal factors of the users and others involved with m-health continuance. The important manageable factors included trust and the doctor’s ability and benevolence. These should be integrated into an improvement plan of m-Health services.

This research contributes to the literature in three ways. First, by using the S-O-R framework, this study explores both the trust and the risk factors of the three major actors related to m-Health services. It provides new evidence on m-Health services, considering the interrelationships among the personal traits, doctor characteristics, perceived risks, cognitive and emotional trust, and two kinds of user post-adoption behavioral intentions [10,53,105] and helping in addressing the human side of m-Health operations [1]. Second, this study advances the existing literature on the investigation of the antecedents and consequences of trust in the m-Health service context. This research considers trust as a multidimensional construct [51] in that comprises three key actors (i.e., individual users, doctors, and platforms) in providing m-Health services. We have examined the distinct roles of cognitive trust and affective trust in m-Health applications, supporting the argument that each type of trust has a different level of impact on user behavioral intention. This study may provide new evidence to support the platform literature [20] on m-Health services [10,105,106]. Finally, our study contributes to the sustainable development of m-Health [1] by suggesting new ways to encourage online consultation during the pandemic. To improve trust in the m-Health platform, the platform can develop the m-Health doctor’s ability and benevolence while reducing the its perceived privacy and physical risks. These are new findings for the sustaining of the m-Health business.

This study provides three practical implications for the promotion of m-Health services in pandemic situations. First, we show that personal traits (i.e., propensity to trust) play a crucial role in developing trust in m-Health platforms [57]. So, the m-Health platform managers may target the m-Health service towards those with a higher propensity to trust in order to expand the user base and develop loyal users. Second, our results show that the doctors’ abilities and benevolence play a vital role in the trust of the m-Health platforms. Thus, this study suggests that while m-Health practitioners should provide cues (e.g., the positions, pictures, and titles of doctors, and the level of hospitals) on the platform regarding the doctors’ abilities in order to develop cognitive trust in the initial phase of a relationship [107], it is more important to cultivate emotional trust through the doctors’ benevolence (e.g., concern for the patients and goodwill for others and a kind attitude) during the interaction with users [10]. Thus, for sustaining the m-Health platform, the m-Health platform providers may specifically recruit benevolent doctors or provide benevolence-related training to the existing ones to encourage more benevolent behaviors in the online consultations via the m-Health platform. Lastly, the results show that the perceived privacy and physical risks of the m-Health platform reduce both emotional and cognitive trust in the m-Health platform. The m-Health service providers might address this issue by developing new protocols for physical safety guarantees [16,58,68,108]. Moreover, the platform might protect user privacy by publishing and enforcing privacy regulations [21,109].

This study has several limitations to be considered in future research. First, this study only considered m-Health users’ post-behavioral intentions, but not their actual reuse behaviors. Future research may consider how to use dynamic tracking methods to obtain the actual behaviors of individual users if the data privacy problem can be resolved [110]. Second, we used only cognitive and emotional trust to explore the internal linkage mechanisms in the S-O-R framework. There could be other possible mediators, such as satisfaction and motivation [26,27], which require further studies. Third, as our survey respondents are mainly young to middle-aged, our study is limited in the assessment of the m-Health needs of the elderly patients and those of the chronically ill and might have a selection bias with regard to the patients with different ages. For example, Tian and Wu [95] notes that the elderly patients with chronic diseases are particularly concerned about the performance expectancy and the social influence on the others. While the existing literature suggests that a majority of the m-Health users in China are relatively young to middle-aged adults, future research may target the elderly patients to replicate our study. Fourth, we did not include other possible antecedents (e.g., IT characteristics, user health conscience, health status, etc.) in this study, which might influence users’ post-adoption behaviors [111,112,113]. The future research may address this issue by examining those antecedents and mediators. Finally, as a cross-sectional study, this study is limited to explaining the causal relationships in the model. The future research may conduct experiments or longitudinal studies to verify our findings.

## Figures and Tables

**Table 1:** Profiles of key m-Health platforms for online consultations.

*Platforms*	*Haodf.com*	*WeDoctor*	*Chunyu Doctor*	*Ping An Good Doctor*	*AliHealth*	*JD Health*
***Headquarters***	*Beijing*	*Hangzhou*	*Beijing*	*Shanghai*	*Beijing*	*Beijing*
***Year founded***	*2006*	*2010*	*2011*	*2014*	*2004*	*2017*
***Ownership***	*Private*	*Private*	*Private*	*Publicly listed*	*Publicly listed*	*Publicly listed*
***Main functionalities***	*Online consultation,* *Sales of medicines*	*Online consultation,* *Sales of medicines and insurance*	*Online consultation,* *Sales of medicines*	*Online consultation,* *Sales of medicines* *Health management programs*	*Online consultation,* *Sales of medicines, health products and insurance* *Health management programs*	*Online consultation,* *Sales of medicines, health products and insurance* *Health management programs*

Adapted from Cheng et al., 2022 [83].

**Table 2:** Sample profiles.

Variables	Level	Frequency	Percent
Gender	Female	379	55.8
	Male	300	44.2
Age	18–25	120	17.7
	26–30	175	25.8
	31–40	305	44.9
	41–50	59	8.7
	>50	20	2.9
Marital status	Single	162	23.9
	Married	517	76.1
Salary	Less than RMB 3000	37	5.4
	RMB 3000~RMB 4999	60	8.8
	RMB 5000~RMB 7999	196	28.9
	RMB 8000~RMB 9999	186	27.4
	More than RMB 10,000	200	29.5
Education	Less than high school degree	11	1.6
	College graduate or student	54	8.0
	Undergraduate or student	533	78.5
	Masters postgraduate degree or above	81	11.9
Apps	Ping An Good Doctor	308	45.4
	Good Doctor	143	21.1
	Wei Mai	3	0.4
	Wei Yi	32	4.7
	Spring Rain Doctor	55	8.1
	Ding Xiang Doctor	137	20.2
	Others	1	0.1
Frequency	≤1 time	97	14.3
	2 times–3 times	392	57.7
	4 times–5 times	135	19.9
	≥6 times	55	8.1

**Table 3:** Results of the measurement model.

Constructs	Items	Factor Loadings	Cronbach’s Alpha	CR	AVE
Propensity to trust	DispositionToTrust1	0.869 ***	0.883	0.919	0.74
	DispositionToTrust2	0.89 ***			
	DispositionToTrust3	0.874 ***			
	DispositionToTrust4	0.806 ***			
Doctor’s ability	Ability1	0.737 ***	0.693	0.813	0.521
	Ability2	0.666 ***			
	Ability3	0.718 ***			
	Ability4	0.762 ***			
Doctor’s benevolence	Benevolence1	0.733 ***	0.758	0.846	0.579
	Benevolence2	0.807 ***			
	Benevolence3	0.766 ***			
	Benevolence4	0.735 ***			
Privacy risk	PrivacyRisk1	0.924 ***	0.916	0.947	0.856
	PrivacyRisk2	0.917 ***			
	PrivacyRisk3	0.934 ***			
Physical risk	PhysicalRisk1	0.77 ***	0.81	0.875	0.638
	PhysicalRisk2	0.856 ***			
	PhysicalRisk3	0.794 ***			
	PhysicalRisk4	0.77 ***			
Cognitive trust	CognitionbasedTrust1	0.707 ***	0.691	0.812	0.519
	CognitionbasedTrust2	0.697***			
	CognitionbasedTrust3	0.713 ***			
	CognitionbasedTrust4	0.763 ***			
Emotional trust	AffectbasedTrust1	0.691 ***	0.734	0.834	0.556
	AffectbasedTrust2	0.761 ***			
	AffectbasedTrust3	0.74 ***			
	AffectbasedTrust4	0.789 ***			
Continuance intention	ContinuanceIntention1	0.732 ***	0.704	0.818	0.529
	ContinuanceIntention2	0.707 ***			
	ContinuanceIntention3	0.729 ***			
	ContinuanceIntention4	0.741 ***			
Positive WOM	PositiveWOM1	0.796 ***	0.79	0.864	0.615
	PositiveWOM2	0.82 ***			
	PositiveWOM3	0.707 ***			
	PositiveWOM4	0.81 ***			

Note: *** *p*-value < 0.001.

**Table 4:** Results of the discriminant validity (cross-loading criterion).

	Propensity to Trust	Ability	Benevolence	Privacy Risk	Physical Risk	Cognitive Trust	Emotional Trust	Continuance Intention	Positive WOM
DispositionToTrust1	0.869	0.124	0.192	−0.119	−0.112	0.216	0.216	0.112	0.192
DispositionToTrust2	0.89	0.161	0.201	−0.101	−0.159	0.263	0.228	0.186	0.214
DispositionToTrust3	0.874	0.161	0.186	−0.149	−0.164	0.293	0.251	0.167	0.201
DispositionToTrust4	0.806	0.178	0.134	−0.071	−0.148	0.218	0.227	0.212	0.217
Ability1	0.127	0.737	0.458	−0.172	−0.262	0.455	0.357	0.397	0.353
Ability2	0.178	0.666	0.448	−0.213	−0.295	0.348	0.322	0.299	0.296
Ability3	0.094	0.718	0.373	−0.196	−0.243	0.428	0.349	0.391	0.391
Ability4	0.134	0.762	0.47	−0.225	−0.256	0.456	0.365	0.351	0.381
Benevolence1	0.149	0.397	0.733	−0.221	−0.222	0.401	0.421	0.302	0.351
Benevolence2	0.172	0.488	0.807	−0.251	−0.243	0.486	0.449	0.305	0.385
Benevolence3	0.156	0.455	0.766	−0.226	−0.269	0.491	0.457	0.295	0.406
Benevolence4	0.155	0.502	0.735	−0.215	−0.238	0.422	0.383	0.28	0.324
PrivacyRisk1	−0.134	−0.257	−0.274	0.924	0.465	−0.332	−0.357	−0.338	−0.343
PrivacyRisk2	−0.117	−0.266	−0.293	0.917	0.457	−0.338	−0.4	−0.313	−0.358
PrivacyRisk3	−0.11	−0.248	−0.268	0.934	0.44	−0.351	−0.396	−0.331	−0.366
PhysicalRisk1	−0.148	−0.215	−0.211	0.441	0.77	−0.292	−0.289	−0.285	−0.302
PhysicalRisk2	−0.157	−0.362	−0.317	0.431	0.856	−0.387	−0.339	−0.395	−0.408
PhysicalRisk3	−0.15	−0.289	−0.26	0.339	0.794	−0.373	−0.319	−0.378	−0.318
PhysicalRisk4	−0.085	−0.279	−0.222	0.362	0.77	−0.308	−0.274	−0.352	−0.299
CognitionbasedTrust1	0.226	0.464	0.494	−0.26	−0.288	0.707	0.471	0.398	0.439
CognitionbasedTrust2	0.226	0.416	0.415	−0.275	−0.313	0.697	0.459	0.364	0.4
CognitionbasedTrust3	0.206	0.404	0.392	−0.253	−0.31	0.713	0.449	0.368	0.437
CognitionbasedTrust4	0.184	0.411	0.41	−0.274	−0.328	0.763	0.431	0.448	0.523
AffectbasedTrust1	0.146	0.337	0.401	−0.271	−0.293	0.47	0.691	0.319	0.416
AffectbasedTrust2	0.23	0.294	0.381	−0.388	−0.295	0.443	0.761	0.369	0.463
AffectbasedTrust3	0.205	0.44	0.5	−0.278	−0.254	0.467	0.74	0.392	0.486
AffectbasedTrust4	0.217	0.363	0.394	−0.306	−0.307	0.492	0.789	0.388	0.503
ContinuanceIntention1	0.149	0.419	0.305	−0.23	−0.323	0.436	0.377	0.732	0.435
ContinuanceIntention2	0.115	0.341	0.232	−0.189	−0.257	0.343	0.294	0.707	0.359
ContinuanceIntention3	0.192	0.346	0.315	−0.283	−0.31	0.429	0.4	0.729	0.405
ContinuanceIntention4	0.111	0.342	0.267	−0.322	−0.399	0.379	0.355	0.741	0.376
PositiveWOM1	0.18	0.365	0.383	−0.289	−0.332	0.498	0.523	0.453	0.796
PositiveWOM2	0.204	0.403	0.411	−0.317	−0.329	0.498	0.524	0.398	0.82
PositiveWOM3	0.15	0.397	0.347	−0.287	−0.303	0.475	0.415	0.423	0.707
PositiveWOM4	0.214	0.387	0.375	−0.314	−0.349	0.495	0.502	0.433	0.81

**Table 5:** Results of discriminant validity (Fornell and Larcker criterion).

	Doctor’s Ability	Doctor’s Benevolence	Cognitive Trust	Continuance Intention	Emotional Trust	Physical Risk	Positive WOM	Privacy Risk	Propensity to Trust
Doctor’s ability	**0.722**								
Doctor’s benevolence	0.605	**0.761**							
Cognitive trust	0.588	0.594	**0.721**						
Continuance intention	0.5	0.388	0.55	**0.727**					
Emotional trust	0.483	0.563	0.627	0.494	**0.746**				
Physical risk	−0.363	−0.32	−0.43	−0.444	−0.384	**0.798**			
Positive WOM	0.494	0.484	0.627	0.544	0.628	−0.419	**0.784**		
Privacy risk	−0.278	−0.301	−0.368	−0.354	−0.416	0.49	−0.385	**0.925**	
Propensity to trust	0.182	0.208	0.291	0.198	0.269	−0.171	0.239	−0.13	**0.86**

**Table 6:** Results of discriminant validity (HTMT criterion).

	Doctor’s Ability	Doctor’s Benevolence	Cognitive Trust	Continuance Intention	Emotional Trust	Physical Risk	Positive WOM	Privacy Risk	Propensity to Trust
Doctor’s ability									
Doctor’s benevolence	0.837								
Cognitive trust	0.845	0.817							
Continuance intention	0.709	0.527	0.778						
Emotional trust	0.674	0.751	0.883	0.679					
Physical risk	0.482	0.403	0.57	0.581	0.497				
Positive WOM	0.667	0.623	0.846	0.726	0.821	0.519			
Privacy risk	0.351	0.36	0.463	0.438	0.507	0.572	0.453		
Propensity to trust	0.235	0.253	0.37	0.246	0.331	0.198	0.286	0.143	

**Table 7:** Results of Path Coefficients.

	β	STDEV	T Values	*p* Values	Status
H1a Propensity to trust -> Cognitive trust	0.134	0.029	4.637	0.000	Accepted
H1b Propensity to trust -> Emotional trust	0.125	0.034	3.693	0.000	Accepted
H2a Doctor’s ability -> Cognitive trust	0.293	0.04	7.258	0.000	Accepted
H2b Doctor’s ability -> Emotional trust	0.158	0.041	3.838	0.000	Accepted
H3a Doctor’s benevolence -> Cognitive trust	0.31	0.039	7.904	0.000	Accepted
H3b Doctor’s benevolence -> Emotional trust	0.351	0.042	8.371	0.000	Accepted
H4a Privacy risk -> Cognitive trust	−0.102	0.032	3.212	0.001	Accepted
H4b Privacy risk -> Emotional trust	−0.206	0.035	5.869	0.000	Accepted
H5a Physical risk -> Cognitive trust	−0.151	0.034	4.431	0.000	Accepted
H5b Physical risk -> Emotional trust	−0.092	0.039	2.366	0.018	Accepted
H6a Cognitive trust -> Continuance intention	0.395	0.042	9.471	0.000	Accepted
H6b Cognitive trust -> Positive WOM	0.299	0.042	7.111	0.000	Accepted
H7a Emotional trust -> Continuance intention	0.246	0.041	5.989	0.000	Accepted
H7b Emotional trust -> Positive WOM	0.334	0.043	7.794	0.000	Accepted
H8 Continuance intention -> Positive WOM	0.214	0.044	4.919	0.000	Accepted

**Table 8:** Results of R^2^ and adjusted R^2^.

	R Square	R Square Adjusted
Cognitive trust	0.498	0.494
Emotional trust	0.428	0.424
Continuance intention	0.339	0.337
Positive WOM	0.514	0.512

**Table 9:** Results of f^2^ value.

	Cognitive Trust	Emotional Trust	Continuance Intention	Positive WOM
Propensity to trust	0.034	0.026		
Ability	0.102	0.026		
Benevolence	0.116	0.13		
Cognitive trust			0.143	0.098
Emotional trust			0.056	0.132
Privacy risk	0.015	0.054		
Physical risk	0.032	0.01		
Continuance intention				0.062
Positive WOM				

**Table 10:** Results of Q^2^.

	SSO	SSE	Q^2^ (=1-SSE/SSO)
Propensity to trust	2716	2716	
Ability	2716	2716	
Benevolence	2716	2716	
Privacy risk	2037	2037	
Physical risk	2716	2716	
Cognitive trust	2716	2027.111	0.254
Emotional trust	2716	2084.539	0.232
Continuance intention	2716	2240.208	0.175
Positive WOM	2716	1868.289	0.312

## Data Availability

The data presented in this study are available on request from the corresponding author. The data are not publicly available due to privacy.
